# Lignocellulosic Based Biochar Adsorbents for the Removal of Fluoride and Arsenic from Aqueous Solution: Isotherm and Kinetic Modeling

**DOI:** 10.3390/polym14040715

**Published:** 2022-02-12

**Authors:** Iram Ayaz, Muhammad Rizwan, Jeffery Layton Ullman, Hajira Haroon, Abdul Qayyum, Naveed Ahmed, Basem H. Elesawy, Ahmad El Askary, Amal F. Gharib, Khadiga Ahmed Ismail

**Affiliations:** 1 US Pakistan Center for Advanced Studies in Water, Mehran University of Engineering and Technology, Jamshoro 76062, Pakistan; iree.az308@gmail.com (I.A.); naveed.uspcasw@faculty.muet.edu.pk (N.A.); 2 Department of Civil and Environmental Engineering, University of Utah, 201 Presidents Circle, Room 201, Salt Lake City, UT 84112, USA; 3 Rwanda Institute of Conservation Agriculture, Kagasa-Batima Rd, Gashora, Bugesera, Rwanda; 4 Department of Environmental Sciences, The University of Haripur, Haripur 22620, Pakistan; hajira@uoh.edu.pk; 5 Department of Agronomy, The University of Haripur, Haripur 22620, Pakistan; 6 Department of Pathology, College of Medicine, Taif University, P.O. Box 11099, Taif 21944, Saudi Arabia; basemelesawy2@gmail.com; 7 Department of Clinical Laboratory Sciences, College of Applied Medical Sciences, Taif University, P.O. Box 11099, Taif 21944, Saudi Arabia; ahmedelaskary3@gmail.com (A.E.A.); dr.amal.f.gharib@gmail.com (A.F.G.); khadigaah.aa@tu.edu.sa (K.A.I.)

**Keywords:** kinetic study, *Eucalyptus*, biochar, arsenic, fluoride

## Abstract

*Eucalyptus* wood is made up of lignocellulosic material; this lignocellulosic material contains two types of biopolymers, i.e., carbohydrate and aromatic polymers. In this study, this lignocellulosic material was used to prepare biochar. Three biochar, i.e., laboratory-based (B1), barrel-based (B2), and brick kiln-biochar (B3), were used for fluoride and arsenic removal from aqueous solution. Barrel-based biochar was prepared by using the two-barrel method’s alteration. The highest fluoride removal (99%) was attained at pH 2 in the presence of B1, while in the presence of B2 and B3, maximum fluoride removal was 90% and 45.7%, respectively. At pH 10, the maximum arsenic removal in the presence of B1, B2, and B3 was 96%, 94%, and 93%, respectively. The surface characteristics obtained by Fourier-transform infrared spectroscopy (FTIR) showed the presence of carbonyl group (C-O), and alkene (C=C) functional groups on all the three studied biochars. Isotherm studies showed that the adsorption was monolayered (all the adsorbed molecules were in contact with the surface layer of the adsorbent) as the Langmuir isotherm model best fits the obtained data. Adsorption kinetics was also performed. The R^2^ value supports the pseudo-second-order kinetics, which means that chemisorption was involved in adsorbing fluoride and arsenic. It is concluded that B1 gives maximum removal for both fluoride (99%) and arsenic (96%). The study shows that lignocellulose-based biochar can be used for arsenic and fluoride removal from water.

## 1. Introduction

The presence of toxic substances in drinking water can cause a risk to the human health [1,2]. Pakistan Council of Research in Water Resources (PCRWR) monitored the water quality in some major cities of Pakistan. The study found the presence of arsenic, fluoride, and bacteria in drinking water [3].

Clean water is a fundamental human right, but pollution with metals, non-metals, natural processes, and some inorganic components such as fluoride and arsenic poses serious health problems. Arsenic is an element found in a natural setting, organisms, soil, and aquatic environment. Arsenic enters into the soil and water by biological processes, volcanos, rocks weathering, mining, pesticides with arsenic, and burning fossil fuels [4]. The permissible limit of arsenic in air, freshwater, soil, and seawater is 3 ng m^−3^, 10 mg L^−1^, 100 mg kg^−1,^ and 1.5 mg L^−1^, respectively [5]. Arsenic is among the prominent four non-essential elements: toxic arsenic, mercury, cadmium, and lead. It is also considered the potential carcinogenic element [6]. According to the WHO, it is among group 1 human carcinogens [7].

The well-known active pollutant found in water in numerous areas in Pakistan is fluoride; this is a threat to water quality consumed by the masses. The permissible limit of fluoride in drinking water is 0.5–1.5 mg L^−1^. Unfortunately, 260 million people drink fluoride contaminated (>1.5 mg L^−1^) water. Fluoride is essential for teeth and bones to prevent tooth decay and protect bones. However, a higher fluoride concentration can result in dental and skeletal fluorosis [8]. The general issues of drinking water containing fluoride results in fluorosis, teeth mottles, bones weakness, and it also affects the human’s nervous system [1]. These diseases primarily affect children, who are more susceptible to fluoride than grownups.

Thus, removing these harmful components from water is essential to make it potable. Many available techniques are already used for this purpose: ion exchange, reverse osmosis (RO), and coagulation. The drawbacks of using these methods are their high operation and maintenance costs [9,10,11,12,13,14].

Another emergent technique is known globally; biochar is an active adsorbent to decontaminate various pollutants from water. Biochar is a carbonaceous compound synthesized by pyrolyzing various biomasses, i.e., wood, leaves, vegetable wastes, and seeds [15,16,17].

Adsorption is a process well known for its cost-effectiveness and ability to remove metals and non-metals from water [18]. Adsorption is defined as the adhesion of adsorbate on the surface of the adsorbent, either by physical or chemical adsorption. The main features of biochar are its inorganic constituents, functional groups, pores on its surface, and greater surface area; thus, it can be used as an effective adsorbent material [19,20]. This active material can be produced from various renewable resources [21]. Cocos-nucifera core, pinewood and pine bark, *eucalyptus* bark, and tea waste are the raw materials on which several studies are already conducted to prepare biochar [22,23,24,25].

In this study, biopolymer lignocellulosic material, i.e., eucalyptus wood was used to prepare biochar under three different conditions. These biochars were then evaluated as an an effective adsorbent for fluoride and arsenic removal from synthetic water. This is the first time that three different methods were employed for the preparation of biochar from eucalyptus wood and were compared to remove two pollutants, i.e., arsenic and fluoride from water. Previously, there was no study in which this type of comparison was done. Isotherm and kinetics were also carried out in the current study.

## 2. Materials and Methods

### 2.1. Preparation of Biochars

Laboratory, barrel, and brick kiln biochar were prepared by using branches of *Eucalyptus* plant. Laboratory-based biochar (B1) was prepared in a muffle furnace (Nabertherm B 180, Lilienthal, Germany) in presence of nitrogen gas, barrel-based biochar (B2) was prepared by using the two-barrel method, and the brick kiln biochar (B3) was synthesized in a brick clamp kiln [26,27]. For the preparation of B2 and B3, the branches were cut into 20 cm lengths and chopped using a hand hatchet to obtain a cross-sectional dimension of 1 cm or less. The temperatures used for the preparation of B1, B2, and B3 were 350 °C, 550 °C, and 450 °C, respectively. For B1, the split branches were cut into the size of 1 cm length. A 2.5 L airtight, stainless-steel reactor with incorporated vents for air inflow and outflow was used to prepare B1. Stainless-steel reactor was put inside a muffle furnace, and the temperature was recorded as 350 °C for 2 h. Nitrogen was continuously purged during the experiment to guarantee an oxygen-free environment. After the preparation of biochar, it was crushed by using a mortar and pestle. The biochar was sieved using 0.595 mm^−1^ mm sizes and saved in zip lock bags for further batch experimentation.

### 2.2. Characterization of Biopolymer Containing Biochar

SEM (scanning electron microscope) was used to generate an image of the material under study with varying magnification. Brunauer–Emmett–Teller (BET) was used to determine biochars surface area. Zeta potential technique was used to calculate the charge on the biochar surface. At the same time, Fourier-transform infrared (FTIR) spectroscopy was used to find the functional groups present on the biochar surface.

### 2.3. Chemicals and Reagents

Stock solution (1000 mg/L) of fluoride was prepared by using 2.2101 g of sodium fluoride (NaF) in a deionized (DI) water. The arsenic solution was prepared by using a 1000 ppm standard solution of arsenic.

The solution’s pH was adjusted using 0.1 M NaOH and 0.1 M NaCl solutions.

### 2.4. Analysis

Fluoride was analyzed by using SPANDS method and UV-spectrophotometer (Perkinelmer model: lamda 365, Waltham, MA, USA). Arsenic was analyzed by using inductively coupled plasma mass spectrometry (ICPMS) (Perkinelmer model: Nexion 350, Waltham, MA, USA). All samples were analyzed through standard methods [28,29].

#### 2.4.1. Batch Experiments for Fluoride and Arsenic Adsorption

In these experiments, the adsorption capacity of B1, B2 and B3 biochars were examined. Different concentrations of fluoride and arsenic (10, 30, 45, and 60 mg L^−1^) having 10 mL volume were used with 0.1 g of biochars during the batch experiment. The effect of various parameters like pH, adsorbate concentration, and time on the adsorption of arsenic and fluoride on the three prepared biochars were examined and optimized.

The sorption efficiency or percent removal and sorption capacity were determined by using the following equations:

(1) $$\mathrm{Sorption}\;\mathrm{efficiency}(\%)=\frac{\left(\mathrm{Co}\;-\mathrm{Cf}\right)}{\mathrm{Co}}\;\times \;100$$

(2) $$\mathrm{Sorption}\;\mathrm{capacity}\;(q)=\frac{\left(\mathrm{Co}\;-\mathrm{Ce}\right)}{M}\;\times \;V$$

where q indicates the metal uptake (mg g^−1^), Co and Cf indicate the initial and equilibrium concentrations (mg L^−1^) before and after adsorption, respectively, V indicates the volume of synthetic solution (mL), and M represents the adsorbent dose (g).

Isotherm and Kinetic models:

Freundlich and Langmuir isotherm models were used in the current study. The adsorption isotherms help understand the affiliation among adsorbate concentration and its amount accumulated on the adsorbent surface [30,31,32].

Linear form of Langmuir isotherm was used

(3) $$\frac{\mathrm{Ce}}{\mathrm{qe}}=\frac{1}{Q\;\max\;b}+\frac{\mathrm{Ce}}{Q\;\max}$$

where Ce is the equilibrium concentration, qe is the amount of adsorbent adsorbed, and Qmax and b are known as Langmuir constants linked to adsorption capability and sorption affinity, respectively. The slopes can measure the plot’s Q max and b-intercept in Ce/qe against Ce.

The Freundlich isotherm is

(4) $$\mathrm{Log}\;\mathrm{qe}=\mathrm{Log}\;\mathrm{Kf}+(\frac{1}{n})\log\mathrm{Ce}$$

where qe is the adsorbed quantity of adsorbate at equilibrium, Ce is the equilibrium concentration of adsorbate, Kf is the sorption capacity, and 1/n is the heterogeneity factor.

Pseudo first order and pseudo-second-order kinetic models were applied to find out the adsorption mechanism. The equations used for adsorption kinetics were:

Pseudo First order (PFO):

Ln (qe − qt ) = ln qe − k1t(5)

Pseudo Second-order (PSO):

(6) $$\frac{t}{qt}=\frac{1}{k2\mathrm{qe}2}+\frac{1}{\mathrm{qe}\;}$$

where k1 and k2 are the rate constants of pseudo 1st and pseudo 2nd order, qe is the amount of adsorbate adsorbed at equilibrium, and qt is the amount of adsorbate adsorbed at time t [32,33].

#### 2.4.2. Statistical Analysis

The statistical analysis was carried out using Microsoft excel by incorporating the above equations into the software. The graphs were developed in Sigma plot.

## 3. Results

### 3.1. Characterization

#### 3.1.1. Brunauer Emmet–Teller (BET)

Surface area (SA) is one of the main factors in determining the biochar ability to adsorb various contaminants. The surface area of all three prepared biochars was measured with the help of BET analysis [34]. The B1, B2, and B3 biochars’ surface area was 0.885 ± 0.505, 99.449 ± 9.091, and 6.341 ± 0.427 m^2^/g, respectively (Table 1). B2 biochar had the highest surface area than B1 and B3 and is also greater than many other biochars found in the literature. The surface areas of coffee ground carbon, perilla leaf, dry pinewood, and pine bark biochars were reported to be 5.0, 3.2, 2.73, and 1.88 mm, respectively.

#### 3.1.2. Scanning Electron Microscope Analysis (SEM)

**Figure 1:**
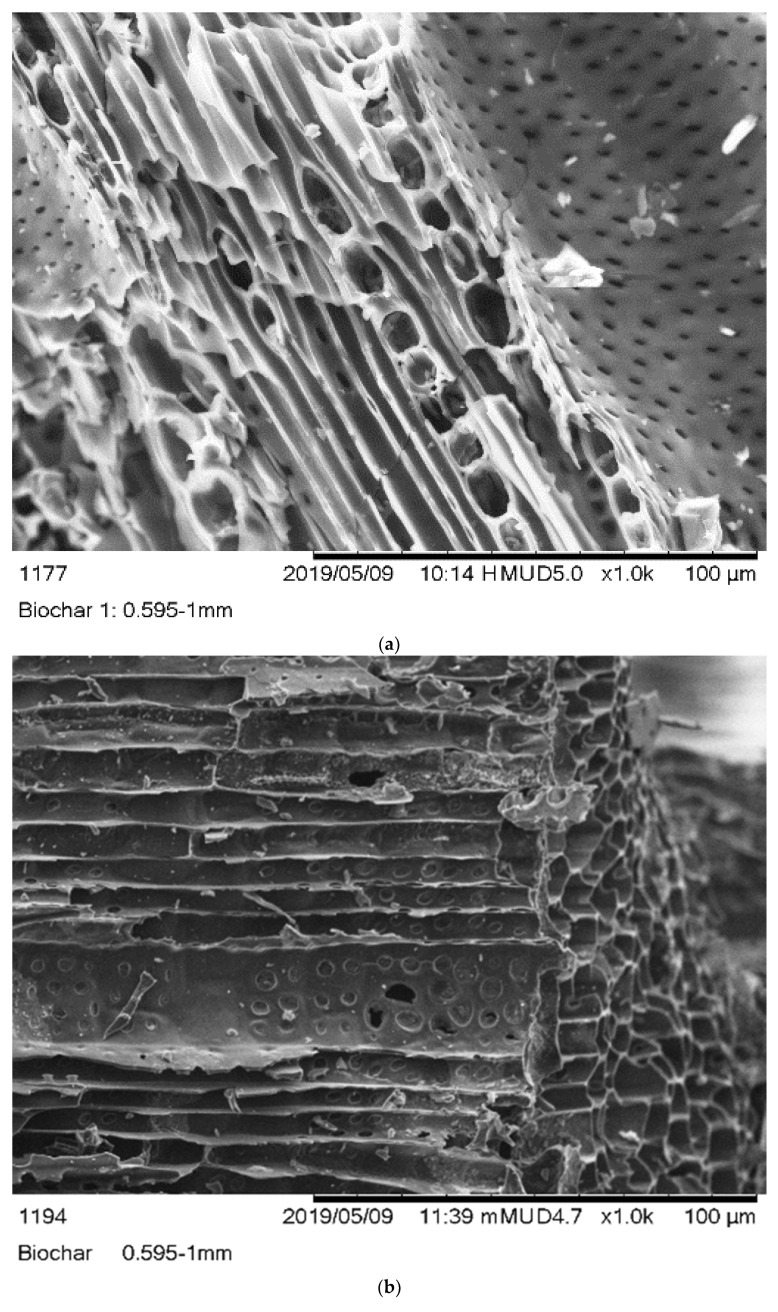

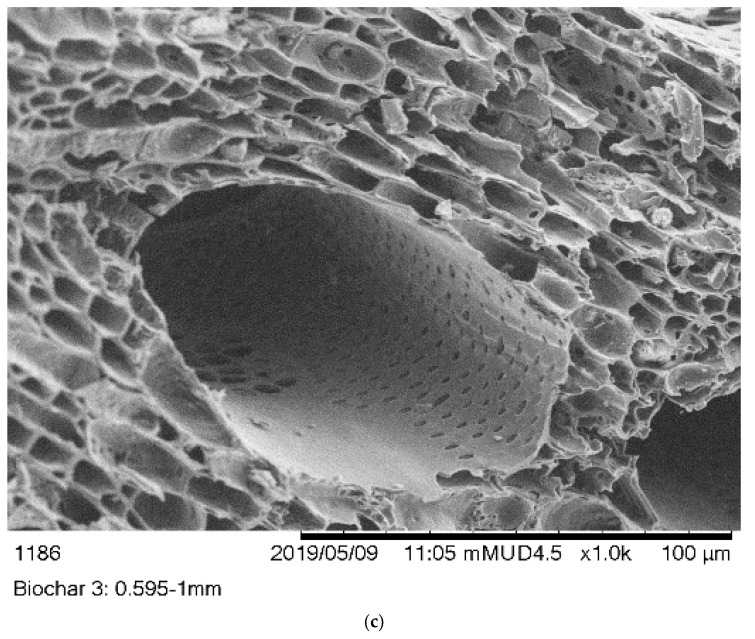
SEM images of (**a**) (B1) Laboratory-biochar, (**b**) (B2) Barrel biochar, and (**c**) (B3) Brick Kiln biochar.

The monographs of B1, B2, and B3 biochars were captured using SEM. Figure 1 showed that the B1 and B2 biochars has a heterogeneous surface with a honeycomb structure. In contrast, the B3 biochar showed a rough and uneven surface. The average pore sizes of B1, B2, and B3 biochars were 0.061, 0.276, and 0.100 µm, respectively.

#### 3.1.3. FTIR

**Figure 2:**
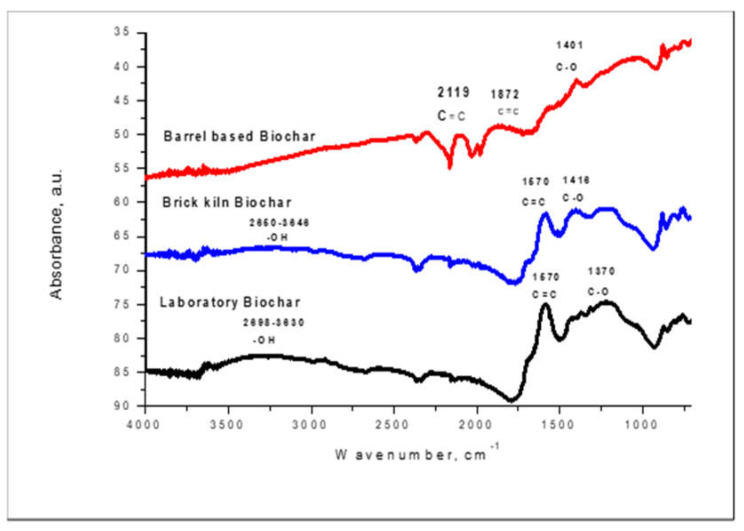
FTIR of Laboratory (B1), Barrel (B2), and Brick Kiln biochar (B3).

The functional groups on the biochars surface were determined using the FTIR method. The FTIR spectroscopy measures the surface chemistry of a solid material. A band of spectra was formed by a range of functional groups present on the surface of prepared biochars. The functional groups present on the surface of B1, B2, and B3 biochars are shown in the Figure 2.

In the case of B1, B2, and B3, the absorbance peaks at 1401 cm^−1^, 1408 cm^−1^, and 1370 cm^−1^ indicated the presence of a C–O functional group.

B1 and B3 contain a C=C functional group at the peaks of 1670 cm^−1^ while B2 contains a C=C functional group at 1872 cm^−1^. Thus, these C–O and C=C functional groups present on the surfaces of B1, B2, and B3 are involved in the adsorption of fluoride and arsenic.

#### 3.1.4. Zeta Potential

Zeta potential gives us information about the surface charges of a material. The B1, B2, and B3 have surface charges of −41.37 mV, −40.30 mV, and −42.67 mV, respectively. These obtained values showed that B2 has more negative zeta potential than biochars prepared by the remaining two methods. This specifies that the surfaces of biochars are considered negative.

### 3.2. Batch Adsorption of F^-^ Fluoride and As-Arsenic

#### 3.2.1. Effect of Contact Time

**Figure 3:**
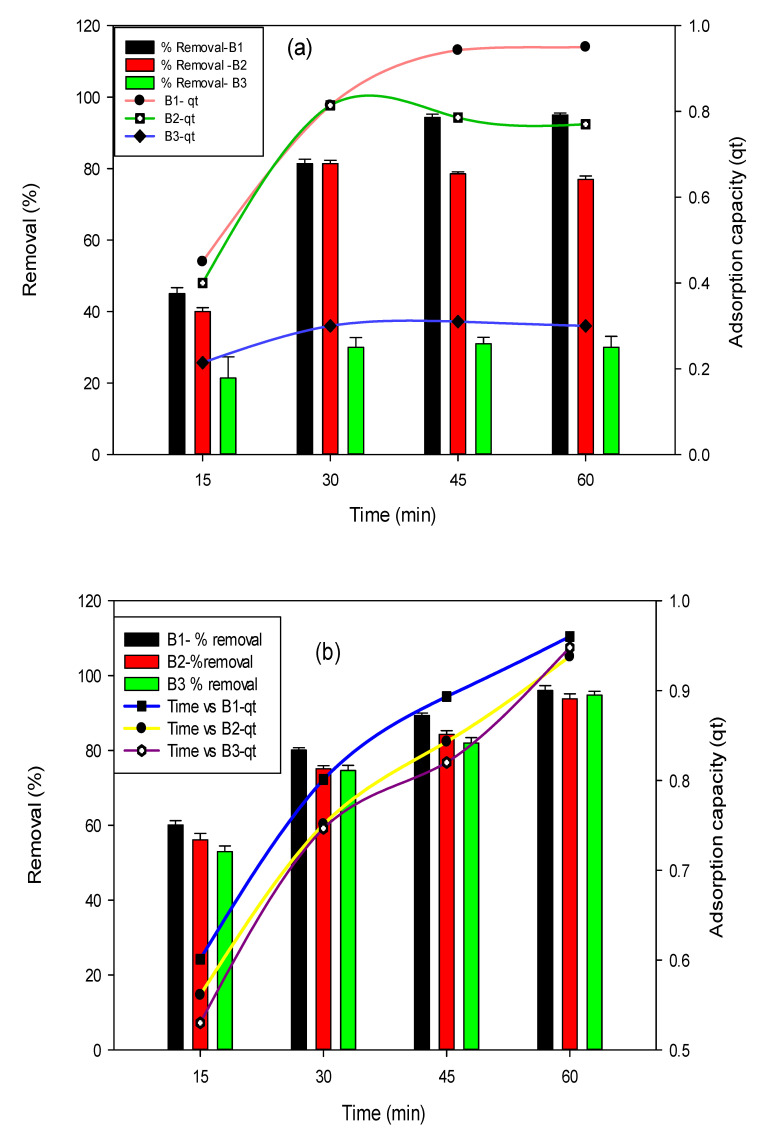
Effect of time on fluoride (**a**) and arsenic (**b**) removal percent and adsorption capacities using B1 (laboratory-based biochar), B2 (Barrel- biochar), and B3 (Brick kiln biochar).

The influence of time was observed in this study by varying the time from 15–60 min, whereas the initial concentration of fluoride and arsenic was kept at 10 mg/L and 0.5 mg/L, respectively. The highest removal efficiency of 95% was observed after 1 h contact time for fluoride with B1 as shown in the Figure 3a. In context with arsenic (Figure 3b), the B1 biochar has shown an adsorption efficiency of 96% in 1 h equilibrium time. At first, generally, rapid uptake of adsorbate occurs because plenty of active binding sites were present on biochars surface. In the case of fluoride removal using B2 and B3, the equilibrium time was attained at 45 min and 1 h, respectively. For arsenic, the maximum adsorption was attained at the contact time of 1h.

#### 3.2.2. Effect of pH

**Figure 4:**
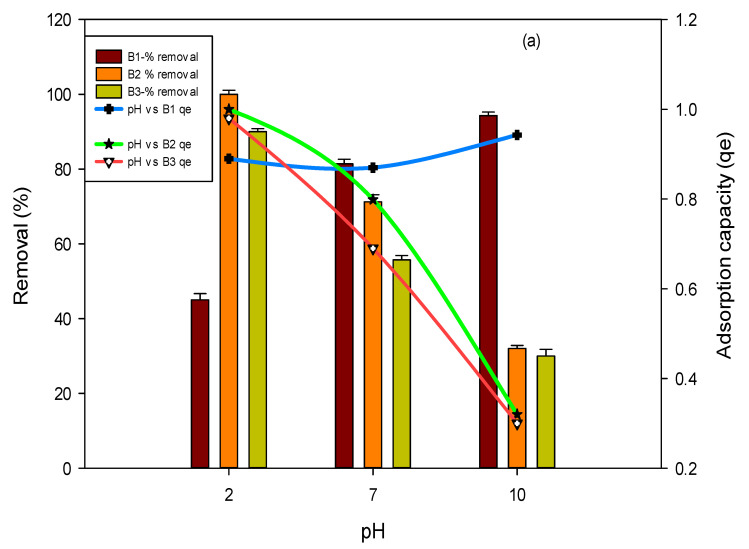

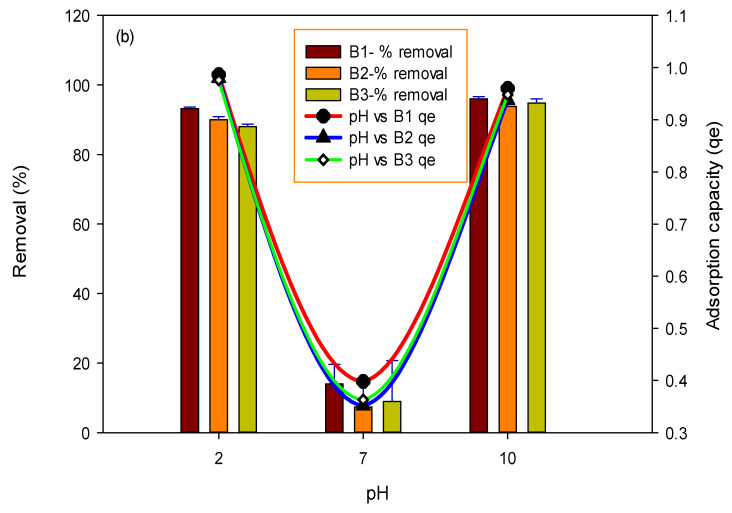
Effect of pH on fluoride (**a**) and arsenic (**b**) removal percent and adsorption capacity by using B1 (laboratory-based biochar), B2 (Barrel- biochar), and B3 (Brick kiln biochar).

One major factor that influences the interaction of biochar with pollutants (fluoride and arsenic) is the solution’s pH. In the current study, the pH of a solution played a vital role in fluoride and arsenic adsorption. The removal of fluoride and arsenic was examined by varying the pH (2–10) of the solution. Results in Figure 4a show that, in the case of B1, a maximum adsorption of 99% was achieved at pH 2 for fluoride, while in the case of B2 and B3, the maximum removal efficiency was calculated to be 90 percent and 45 percent at pH 2 for fluoride. Maximum arsenic removal of 96%, 93% and 94% was achieved at pH 10 for all three studied biochars, i.e., BI, B2 and B3, respectively, as shown in Figure 4b.

#### 3.2.3. Effect of Initial Concentration

**Figure 5:**
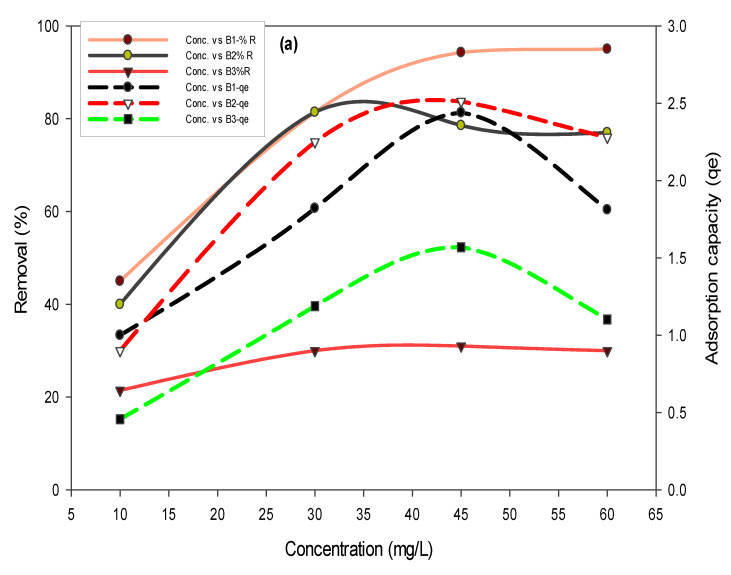

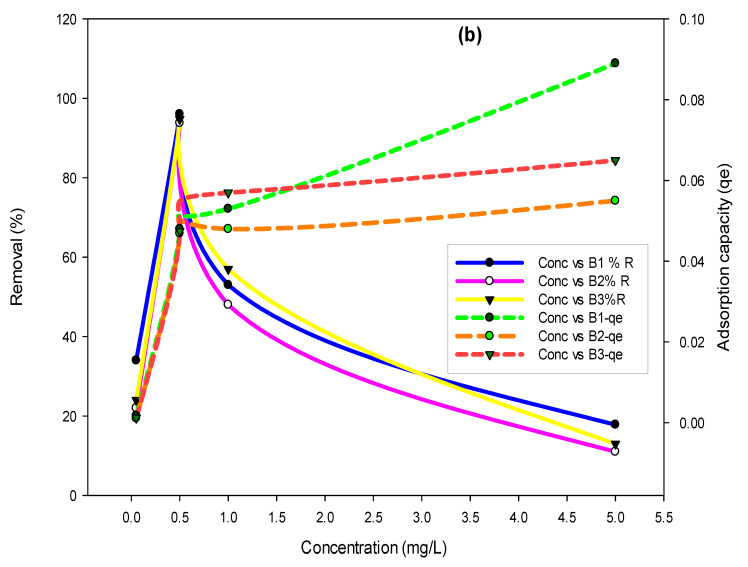
Effect of fluoride (**a**) and arsenic (**b**) concentration on removal percent and adsorption capacities using B1 (laboratory-based biochar), B2 (Barrel- biochar), and B3 (Brick kiln biochar).

To assess the effect of concentration on the removal efficiency of biochars, concentrations of fluoride (10, 30, 45, and 60 mg/L) and arsenic (0.05, 0.51, and 5 mg/L) were varied. Results in Figure 5a showed that the maximum fluoride removal of 99% was achieved in the case of B1, whereas 96% of arsenic removal was achieved (Figure 5b) in the case of B1, where the initial concentration of arsenic was 0.5 mg/L.

### 3.3. Adsorption Isotherms

#### 3.3.1. Isotherms and Kinetic Studies for Fluoride Removal

Langmuir and Freundlich’s models were utilized to check whether the biochar surface is homogeneous or heterogeneous. Table 2 shows that the biochars B1, B2, and B3 prepared with different methods individually were identified as homogenous, because the Langmuir isotherm model fits well with the obtained data. Table 2 shows that for fluoride, the regression coefficient value was highest (0.9937) and maximum sorption capacity (q_max_) was 2.376 in the case of B2. The absence of the heterogeneous nature of biochar was because of deficiency of active areas exponential distribution on its surface. The results showed that fluoride adsorption onto all the three biochars is monolayer because it explains the homogeneous nature of adsorbent and justifies the Langmuir isotherm with the highest R^2^ values.

Moreover, B2 biochar is most appropriate for fluoride adsorption among the three studied biochars. Pseudo first and pseudo second order kinetic models in Table 3 show that the highest R^2^ value (0.9862) is obtained in the case of B3. All three biochars for fluoride favored the pseudo second order rate equation, which revealed the chemisorption nature of all studied biochars for fluoride removal.

#### 3.3.2. Isotherms and Kinetic Studies for Arsenic Removal

Results (R^2^ value) in Table 3 show the homogeneous (monolayer) adsorption on the surfaces of all prepared biochars for arsenic removal. The highest R^2^ value (0.839) for B1 was obtained with arsenic, and its extreme adsorption capacity is 0.108. The B1 biochar is more suitable for arsenic removal from water than B2 and B3.

The kinetic studies data for arsenic removal (Table 3) showed that all prepared biochar in this study best fitted pseudo second order kinetics model. However, B1 has the highest the R^2^ value as compared to B2 and B3 for arsenic removal. This again justifies the chemical adsorption nature of biochars instead of the physical sorption.

## 4. Discussion

In this study, the barrel biochar had a higher surface area (99.449 m^2^/g) than the laboratory and brick kiln biochar. Mishra et al., 2017, reported a *Eucalyptus* biochar with a surface area of 20 m^2^/g which was lower than the surface area of barrel biochar used in the current study [35]. Han et al., 2013, obtained the maximum surface area was 383.66 m^2^/g, achieved in activated softwood biochar [36]. Several factors affect the surface area of biochar; these factors include the type of biomass, pyrolysis temperature, and preparation method. Greater surface area facilitates the adsorption process [37]. Various chars were studied with distinct preparatory methods and concluded that chars with greater surface area, i.e., 55.20 m^2^/g tend to stand out with greater reactivity [27]. However, chars having minimum surface area, i.e., 10.53 m^2^/g, adhere to low reactivity with an adsorbate. Oh et al., 2012, reported deep and variable pore sizes for granular and grounded powder biochar. Moreover, the biochar showed asymmetrical forms and sizes; these sites may provide higher internal surface areas in orange peel and sludge biochar [38].

The morphology of biochar was studied by using SEM technique. The B1 and B2 biochar structures were like a honeycomb, while the surface B3 biochar was rough and uneven. The preparation method of biochar might be the reason for the different morphologies of biochar. In a previous study performed by Hajira et al., 2016, the SEM image of *Eucalyptus camaldulensis* showed a rough and uneven surface with heterogeneous pores of different sizes [39]. Mishra et al., 2017, showed that the biochar prepared from *Eucalyptus* biomass was highly heterogeneous and had macropores [35]. Zhang et al., 2018, used *Eucalyptus* sawdust biochar for chromium removal. The surface of biochar was rough, and it contained heterogeneous particles [40].

Generally, FTIR spectra was observed in the range from 4000 to 500 cm^−1^ [36,41]. The B1 and B3 biochars of the current study showed prominent peaks at absorbance of 1570 cm^−1^, which is characteristic of C=C, and absorbance between 2850–3000 cm^−1^ showed –OH, functional group in both B1 and B3 biochars [42]. All three studied biochars in the current study had a C-O functional group at absorbance peaks of 14416 and 1370 cm^−1^. Biochar B2 has a prominent functional group of alkynes (C≡C) triple bond at an absorbance of 2119 cm^−1^. In a previous study on biochar derived from *Eucalyptus* wood, the FTIR also showed the presence of −COOH, -OH, and C=O functional groups [35]. In another study, perennial grass-based activated biochar was used to remove fluoride and arsenic from an aqueous solution. The FTIR of the activated perennial grass biochar showed the presence of O–H, C-H, C=C, C–O, and -CH functional groups [43]. Papari et al., 2017, used *Conocarpus erectus* biochar for fluoride removal, and the FTIR revealed the presence of following functional groups: ≡C–H and –OH, –CH=CHR, –S=O, C–F, S–OR esters, –C–H and S–S [37]. These functional groups are different due to the biomass used in this study.

The present study achieved the maximum adsorption of fluoride and arsenic within 1 h. Activated rice straw biochar was used to remove fluoride from an aqueous solution. The maximum adsorption was achieved after 3 h [44]. In another study, *Conocarpus erectus* biochar was used by Papari et al., 2017, for maximum fluoride removal from aqueous solution, groundwater, and seawater within 1.5 h [37]. Mishra et al., 2017, used *Eucalyptus* wood for uranium removal in 20 min [35].

The initial concentration of fluoride and arsenic was inversely proportional to the maximum adsorption. A similar trend was observed in a previous study performed by Saikia et al., 2017 in which the increase of fluoride concentration resulted in a decrease in adsorption efficiency [43]. This is due to the saturation of adsorption sites of biochar. Daifullah et al., 2007, also observed that fluoride adsorption increased with the increase in fluoride concentration until the fluoride concentration reached 18 mg/L [44].

As time progresses, removal efficiency declines because of the repulsion between solute constituents on the surface of the adsorbent and in solution [45]. At lower pH, the fluoride removal was recorded to be the highest because, in this condition, the attractive forces were increased amongst biochars and fluoride; this is then due to the H^+^ presence on biochars surface [30]. The phenomenon is known as deprotonation of the functional groups on the adsorbent’s surface if the solution pH was increased, which justifies the arsenic adsorption onto biochars at high pH; this facilitates the adsorption of positively charged ions on the negatively charged surface (biochar) [46]. Moreover, the ion interchange between arsenic and –OH results in maximum arsenic removal at higher pH, i.e., 10 [47]. It is being noticed that if the initial concentration increases, the removal efficiency decreases due to the saturation of the active regions which are present on the biochar [32,44,45].

Likewise, Mohan et al., 2012, used pine wood, and Papari et al., 2017, performed monolayer adsorption experiments for fluoride removal. Langmuir isotherm model was also best fitted to the data in their results [37,48]. In comparison, Goswami et al., 2018, utilized nano-rice husk biochar to remove fluoride and showed that both Langmuir and Freundlich isotherms were best fitted with the its obtained data [33]. These results showed that the interaction of the fluoride particles and biochars is chemical, with the functional groups residing on the biochars surface [31,33]. Biochar made of oak wood, perennial grass, and pine cone [43,49,50] favored monolayer (homogenous) sorption for arsenic, because the adsorption data was well fitted into Langmuir model. There is a possibility that biochars synthesized under higher temperatures have a material with a crystalline structure. This is because of the turbostratic crystallites, which mean biochar has graphene layers ordered not properly [46]. This might be a possible reason for the best fitting of adsorption data with Langmuir model as compared to the Freundlich. A significant R^2^ value was attained [43,45], which also showed a pseudo-second-order rate equation for arsenic removal. Arsenite (As III) and arsenate (As V) were removed from aqueous solution and ground water using perilla leaf biochar. Maximum arsenic removal (88–90%) was achieved at pH 7–9. Langmuir isotherm model was best fitted for both As III and V [49]. Mohan et al., 2014 used magnetic and nonmagnetic stover biochars to remove fluoride from groundwater. Nonmagnetic stover biochar showed better flouride removal capacity while magnetic stover biochar showed better biochar recovery, redispersion and washing [23]. Coffee grounds (CG) were used for the preparation of Carbonaceous material. The carbonaceous coffee grounds were used for the removal of fluoride from water. The CG calcinated at 600 °C showed the maximum fluoride removal compared to the CG calcinated at 400, 800 and 1000 °C [51]. In a study conducted by Papari et al., 2017, *Conocarpus erectus* based granular and powdered biochar was used for fluoride removal from aqueous solution. The maximum fluoride removal in the presence of granular and powdered biochar was 80% and 98.5%, respectively. Langmuir isotherm model was best fitted with the adsorption data [37].

## 5. Conclusions

In the present study, three different types of biochars, i.e., B1, B2, and B3 were prepared from *Eucalyptus* wood. These biochars were then analyzed for their capability/potential for adsorbing both fluoride and arsenic from water. The highest fluoride removal was attained at pH 2, biochar-adsorbent dose of 0.1 g, and a contact time of 60 min in the case of both B1 and B2. For arsenic, maximum removal was obtained at pH 10, 0.1 g (biochar dose), and at a contact time of 60 min using the B1 followed by the B2 biochar. For the B2 biochar, the assessed surface area was 99.449 m^2^/g, and the average pore length was 0.275 µm. The FTIR spectra revealed the involvement of the C-O, and C=C functional group in fluoride adsorption onto B1 and B3. Highest removal of arsenic is attained using the B1 biochar, which revealed that the arsenic might bind to the C-O and C=C functional groups present on the biochar. Langmuir and pseudo second order kinetic models were most suitable for fluoride and arsenic removal for all three studied biochars. The qmax for fluoride was 2.376 mg g^−1^ in the case of B2, whereas the qmax for arsenic was 0.108 mg g^−1^ for B1. Results showed that B1 showed a maximum adsorption of 99% and 96% for both fluoride and arsenic, respectively. It is concluded that cost-effective biopolymer-based biochar could be recommended for the treatment of fluoride and arsenic polluted water.

## Figures and Tables

**Table 1:** Surface area of B1, B2, and B3 biochars.

Adsorbent	Surface Area (m^2^/g)
Laboratory Biochar (B1)	0.885
Barrel Biochar (B2)	99.449
Brick Biochar (B3)	6.341

**Table 2:** Langmuir and Freundlich models’ constants and correlation coefficients, fluoride and arsenic.

Biochar	Adsorbate	Langmuir			Freundlich		
		R^2^	Qmax	b	R^2^	Kf	n
B1	Fluoride	0.977	1.832	0.872	0.756	1.059	5
	Arsenic	0.839	0.1086	1.152	0.339	19.741	2.355
B2	Fluoride	0.993	2.376	1.559	0.835	1.0128	3.593
	Arsenic	0.806	0.066	1.149	0.281	27.646	2.322
B3	Fluoride	0.87	1.333	0.216	0.701	4.149	2.105
	Arsenic	0.776	0.079	1.096	0.284	23.660	2.3164

**Table 3:** Pseudo-first and second-order kinetics for Fluoride and Arsenic.

Biochar	Adsorbate	Pseudo 1st Order			Pseudo 2nd Order		
		R^2^	qe	K1	R^2^	Qe	K2
B1	Fluoride	0.1006	2.027898	0.0276	0.9088	0.123913	69.42557
	Arsenic	0.0469	2.228659	0.0107	0.9733	0.20094	24.70232
B2	Fluoride	0.2866	1.60914	0.0086	0.9249	0.147406	39.30873
	Arsenic	0.0375	2.146632	0.0083	0.9641	0.169739	34.01136
B3	Fluoride	0.1477	12.9656	0.0313	0.9862	0.125	20.20393
	Arsenic	0.0345	2.016776	0.0072	0.9516	0.149301	43.93484

## Data Availability

Data presented in this study are available on fair request to the corresponding author.
